# Nellix Device Failure Mechanisms Analysis on Explanted Grafts

**DOI:** 10.1177/15266028241274736

**Published:** 2024-08-27

**Authors:** Léna Christ, Salomé Kuntz, Damir Vakhitov, Laurent Raibaut, Nicole Neumann, Frédéric Heim, Nabil Chakfé, Anne Lejay

**Affiliations:** 1 GEPROMED, Strasbourg, France; 2 Department of Vascular Surgery, Kidney Transplantation and Innovation, University Hospital of Strasbourg, Strasbourg, France; 3 Vascular Centre, Tampere University Hospital, Tampere, Finland; 4 Biometals and Biological Chemistry group, Institut de Chimie, UMR 7177, CNRS, University of Strasbourg, Strasbourg, France; 5 Laboratory of Textile Physics and Mechanics, Université de Haute-Alsace, Mulhouse, France

**Keywords:** endoprosthesis, aortic aneurysm, degradation, vascular prostheses, endoleaks

## Abstract

**Objective:**

To understand possible reasons for poor durability of the Nellix (Endologix Inc., Irvine, USA) endovascular aneurysm sealing (EVAS) device.

**Materials and Methods:**

21 Nellix endoprostheses explanted for endoleaks and migration underwent visual examinations of stent structures and instrumental examinations of the polymer endobags on 4 devices. We harvested 2.0-gram polymer slices out of each of them and tested the samples in an in vitro implantation replication that included wet and dry exposures. During the wet phase, we placed samples in a beaker with saline, mimicking the filling of the endobags during implantation. An exposure to a 37°C environment with 60% humidity during the dry phase replicated the postimplantation conditions inside the aneurysmal sac.

**Results:**

Iatrogenic defects affected 16 (76%) metal stents and 20 (95%) endobags. The polymer was disintegrated owing to degradation in 15 (71%) cases. The polymer could lose more than 70% of its initial weight when partially dehydrated and regain 80% when placed in saline. We observed volume decrease and polymer fragmentation during these study phases.

**Conclusions:**

The polymer can lose weight and volume while it dehydrates. This structural degradation of the polymer could lead to the development of endoleaks and/or migration of the device.

**Clinical Impact:**

Based on the results of previous investigations, due to possible endovascular device degradation, patients with endografts should be offered life-long surveillance, and the Nellix device is no exception. Herein we suggest polymer degradation as one of the possible reasons for the device failure. Although Nellix has been withdrawn from the market, there are numerous patients with this type of endograft. Due to its unpredictable performance in the medium and long term, these patients should be recommended enhanced life-long surveillance every 6 months. Any suspicious conditions during the follow-up must be taken seriously and explantation should be considered.

## Introduction

Endovascular aneurysm repair (EVAR) has become a routine modality for the treatment of abdominal aortic aneurysms (AAA). Nonetheless, various degradation processes can lead to a loss of stability and structural failure of endografts (EG) over time.^1–3^ Insufficient proximal sealing in a short neck anatomy with type IA endoleaks,^4^ graft thrombosis,^5^ and postprocedural endoleaks of other types similarly remain the reason for multiple additional reinterventions.^6–8^ Type II is the most common and accounts for almost 25% of all endoleak cases.^9^

The Nellix EndoVascular Aneurysm Sealing (EVAS) System (Endologix Inc., Irvine, CA) was supposed to address many of the aforementioned aspects. Its new concept was based on the idea of sealing the entire aneurysmal sac with the integrated biostable polymer endobags, which surrounded 2 balloon-expandable endoprostheses. “Sac anchoring” was a theory to treat AAAs with a new device. It was supposed to diminish the role of conventional neck anatomy, allowing implantations into short aortic necks. The incidence of type II endoleaks was believed to be reduced by the polymer sealing, and the procedure was expected to be simplified and radiation exposure lowered as a result of the shortened peri-procedural time.^10,11^ Despite the initial enthusiasm,^12,13^ EVAS did not meet the expectations. Graft-related failures were identified in over a third of the cases beyond 2 years of follow-up.^14,15^ Migrations, type IA endoleaks, and sac expansions were common and led to secondary aneurysm ruptures or various reinterventions.^15^ The Nellix system was withdrawn from the market in 2019, but numerous patients had been managed with EVAS and require systematic follow-up and, upon necessity, complication-related treatment. Since the failure mechanisms of EVAS are not completely understood, our study aims to analyze and characterize possible mechanisms of their poor durability through a detailed analysis of EVAS devices explanted due to a complication.

## Material and Methods

### Explanted EVAS

From September 2015 to July 2022, EVAS devices from 8 European vascular centers were collected at the laboratory. All received Nellix prostheses were analyzed as part of our vascular explant analysis protocol. Patients’ consents or ethical approval were not required for the examination of explanted devices.

Twenty-one EVAS devices were collected. All of them had been used to treat AAAs. The reasons for explantation were provided by the operating surgeons. They included endoleaks, prosthesis migration, and thrombosis (Table 1). A total of 90.5% (n=19) of the devices were found to have type I endoleaks. Type II endoleaks were reported in 2 cases (9.5%) in combination with type I. A total of 47.6% (n=10) migrations were identified. Nine EVAS devices (42.9%) were characterized to have developed both endoleaks and migrations (Table 1).

**Table 1.** Baseline characteristics of the explanted Nellix (Endologix Inc., Irvine, USA) devices.

			Causes of explantation		
Explant number	Year of implantation	Duration of implantation (months)	Thrombosis	Endoleak (type if any)	Prosthesis migration
1	2014	7	YES	not reported	NO
2	2017	3	NO	I	NO
3	2017	23	NO	I	YES
4	2014	56	NO	IA	YES
5	2015	43	NO	IA	YES
6	2016	38	YES	I	YES
7	2016	40	NO	not reported	YES
8	2015	53	NO	IB & II	NO
9	2013	80	NO	IB & II	NO
10	2015	53	YES	I	NO
11	2015	65	NO	I	NO
12	2017	39	NO	I	NO
13	2014	80	NO	IA	YES
14	2016	56	NO	I	YES
15	2015	70	NO	IA	NO
16	2017	48	NO	I	YES
17	2015	73	NO	I	YES
18	2015	61	NO	I	NO
19	2015	78	NO	IA	YES
20	2016	65	NO	IB	NO
21	2015	85	NO	IA	NO

### Explant Analysis

All EVAS devices were processed and analyzed according to the ISO 9001 protocol, which includes the following steps:

1. Shipping and storage of explants in formalin.
2. Collection of clinical data and anonymous inclusion in a database.
3. Macroscopic examination followed by imaging of segments with a Nikon D5100 camera (Nikon France, Champigny Sur Marne, France).

### Analysis of the Hydrophilic Property of the Polymer

**Figure 1.**
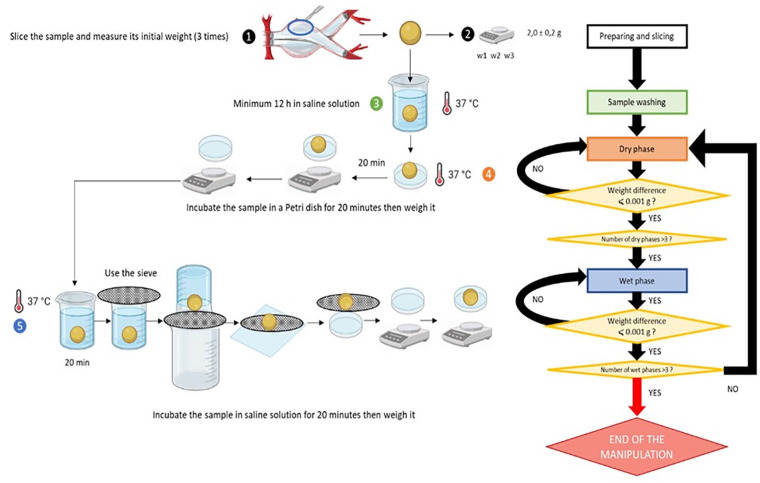
The protocol for studying the hydrophilic properties of the polymer.

Four EVAS devices were randomly selected to easily harvest the polyethylene glycol polymer inside. For each of the 4 endoprostheses, the following protocol was applied (Figure 1).

In brief, the Nellix implantation process includes the deployment of 2 stent-grafts, pre-filling of the endobags around them with saline solution to estimate the polymer volume required. After that the endobags are filled with polymer.^16^ We tried to imitate the polymer implantation process in vitro and study the consequences. We sliced 2.0-gram polymer samples out of the EVAS devices. Each piece was then rinsed with a saline solution to remove the residual formalin in which the explant was stored.

The measurements included wet and dry phases. During the wet phase, the sample was placed in a 25-mL beaker into which 20 mL of saline solution was added. The beaker was placed inside an oven heated to 37°C. The beaker was sealed with parafilm to prevent saline from evaporating. Every 20 minutes, the weight of the sample was evaluated with a Luna Adam LPB analytical balance (Adam Equipment Co Ltd., Kingston, Milton Keynes, UK) with a precision of 0.001 gram. To absorb residual saline, the samples were placed on Whatman paper and then again placed in a 25-mL beaker.

During the dry phase, we placed polymer fragments in a Petri dish on a piece of Whatman paper to absorb liquid residues. The dish was then placed in an oven with a relative humidity of 60% and heated to 37°C, imitating human body conditions. Every 20 minutes, the sample was removed, and its weight was measured. For each sample, 3 cycles of dry and wet phases were performed for demonstration reasons. The stage was considered completed as soon as the tolerated weight difference of 1.0 × 10^-3^grams between at least 2 consecutive measurements was reached.

## Results

### Mechanical Defects

**Figure 2.**
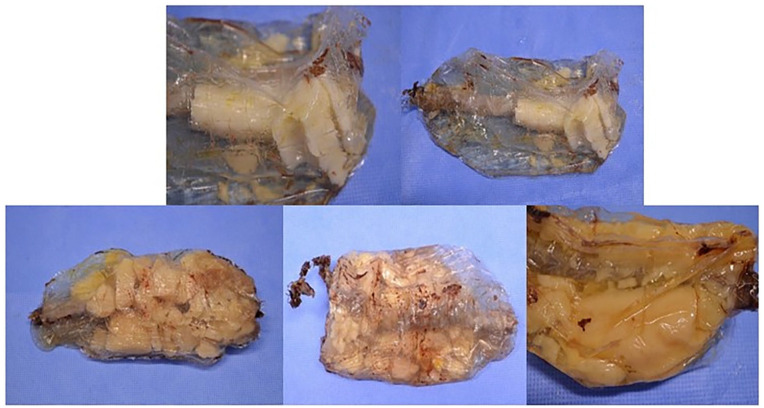
The examples of explanted endobags with fragmented polymer inside.

The median duration of implantation of the devices was 53 months (interquartile range 40 months). The main defects observed in the devices were degradation of the stent metal structure, perforation of the endobags and deterioration of the polymer (Figure 2, Table 2). Degradation of the metal structure, such as breaks in the metal structure or extensions, affected 76% (n=16) of the devices. The characteristics of these deformations, however, suggested they were caused during explantation as footprints of external impact were obvious. Furthermore, 95% (n=20) of the endobags had perforations, with the presence of air and formalin inside. The nature of straight cuts and holes suggested they also occurred during the explantation process. Finally, 71% (n=15) of the endobags contained polymer completely broken into small pieces, which, however, was not caused by explantation when an entire polymer mass or large polymer blocks were present.

**Table 2.** Main deteriorations of the explanted Nellix (Endologix Inc., Irvine, USA) devices.

Explant number	Damaged metal structure	Perforated endobag	Deteriorated polymer
1	No	YES	No
2	YES	YES	No
3	No	YES	YES
4	YES	YES	YES
5	YES	YES	YES
6	YES	YES	YES
7	YES	YES	YES
8	YES	YES	YES
9	YES	YES	No
10	YES	YES	YES
11	YES	YES	No
12	No	YES	No
13	YES	YES	YES
14	YES	YES	YES
15	No	YES	YES
16	YES	YES	YES
17	YES	YES	YES
18	No	YES	YES
19	YES	No	YES
20	YES	YES	YES
21	YES	YES	No

### Polymer Deterioration

The information on the initial weight of each sample is summarized in Table 3.

**Table 3.** The initial data on the studied polymer samples.

Sample	Initial weight (grams)	Causes of explantation	Duration of implantation (months)
A	2.15	Type IA endoleak	56
B	2.03	Type IA endoleak and migration	43
C	2.08	Type IA endoleak and migration	53
D	2.07	Type IA endoleak and migration	72

**Figure 3.**
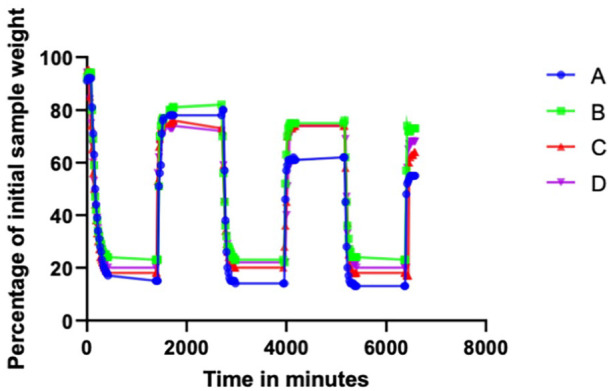
Relative changes of sample weight during the study phases. Data are presented as percentages of the initial sample weight and their relative changes over the experimental time.

**Figure 4.**
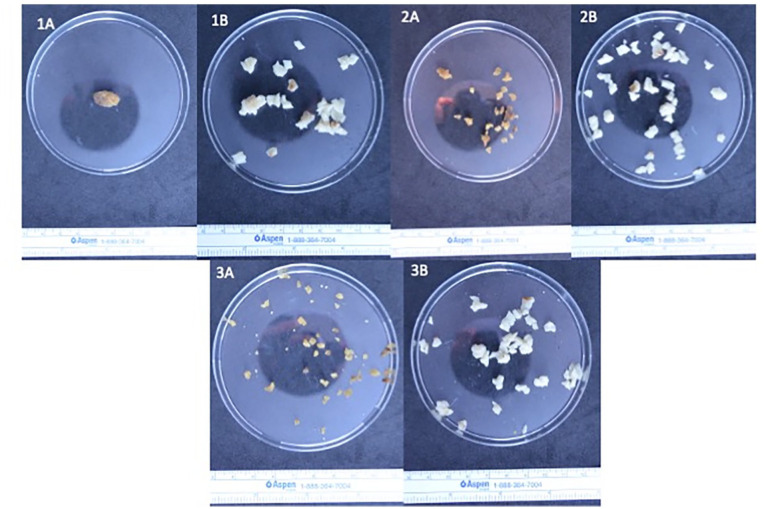
The evolution of a 2.0 ± 0.2 g polymer sample from an explanted Nellix (Endologix Inc., Irvine, USA) device at different experimental stages (1–3) and phases (A—dry phase, B—wet phase).

The weight measurement of the polymer during the 3 cycles of dry and wet phases demonstrated a more than 70% loss of its initial weight during the dry phase. The samples regained 80% of their initial weight when they were immersed in a saline solution (Figure 3, Table 4). Upon visual examination, the polymer demonstrated a volume decrease as well as signs of fragmentation (Figure 4).

**Table 4.** The duration of each phase for each of the 4 samples.

Sample	Dry phase 1 (minutes)	Wet phase 1 (minutes)	Dry phase 2 (minutes)	Wet phase 2 (minutes)	Dry phase 3 (minutes)	Wet phase 3 (minutes)
A	360	230	240	220	240	200
B	340	250	230	220	240	200
C	360	200	220	200	260	160
D	360	220	200	200	240	200
Total time (minutes)	1420	900	890	840	980	760
Mean ± SD (minutes)	355 ± 10	225 ± 21	223 ± 17	210 ± 12	245 ± 10	190 ± 20

Abbreviation: SD, standard deviation.

## Discussion

To our knowledge, this study is among the first few to present detailed information on the possible mechanisms of EVAS failure. In this experimental study, we have demonstrated that the polymer used in the endobags can degrade and lose its consistency.

In comparison with conventional endovascular devices that require appropriate neck sealing, EVAS was supposed to be suitable for a wider range of anatomic presentations without the need for proximal fixation.^15^ Most works, however, fail to explain the possible mechanisms behind the development of complications. In a retrospective study of 280 patients treated with EVAS, therapeutic failure was registered in over 30% of the cases, most of which occurred after 2 years of implantation.^15^ Expectedly, type IA/B endoleaks and stent migrations were significantly associated with sac expansion. In the other series, 161 patients were managed with EVAS and a 43.5% rate of device failure was documented.^17^ Both type IA and type IB endoleaks were common. Sac expansion, caudal stent migrations, stent separations and AAA ruptures were not unusual. The evaluation performed by the same authors stated that “there was no significant difference in failure rates between those cases treated on and off” instructions for use.^17^ In comparison, the post-EVAR type I endoleaks requiring prompt treatment occur in 3 to 15%.^18^ Depending on the series post-EVAR “conversion to open repair can be required in 0.4 to 22% of cases.”^19^ Regardless of any morphologic presentations, the post-EVAS outcomes were worse than the outcomes after conventional endografting, as reported by Quaglino et al.^20^

In the present series, the causes of explantation were in line with the previously published reports.^15,17,21^ Ninety percent were performed due to a type I endoleak. Although the reasons for failure could be multifactorial, the presence of numerous type I endoleaks raises the question of whether the filling of an aneurysmal sac with a polymer is sufficient throughout the whole postoperative period. The possibility of changes in polymer properties as well as the physio-chemical properties of aging must be emphasized here. In our experiment, we tried to imitate the intraoperative and postoperative conditions. As a result, the polymer preserved its hydrophilic properties but failed to retain its weight, volume and shape, undergoing fragmentation when the material partially dehydrated as cross-linked hydrogel was permeable and shrank over time. Upon implantation, the volume of the injected polymer is measured using a saline solution. The polymer is then injected into the endobag. Finally, the polymer is polymerised in situ. This process can lead to errors in polymer volume or induce nonhomogeneous polymerisation, ie, nonhomogeneous polymer mix/problems with polymer cross-linking during polymerisation. As a result, the final polymer will not be sufficiently watertight and will be less resistant to deformation stress as soon as the device is installed. This finding can well explain the instability of implanted devices, since their “anchoring” effect disappears. Consequently, this condition opens the door to endoleaks. The inability to detect or difficulties in detecting post-EVAS endoleaks, particularly in the early stage, have been acknowledged.^22,23^ If the endoleak is left untreated, subsequent sac expansion and pressurization may lead to further morphologic changes in an aneurysm and result in stent migration and/or AAA rupture. The continuous rhythmic movements of the aorta and device could increase the altered area over time and consequently result in the instability and loss of aneurysm sealing.

Our work has an important clinical implication. Careful monitoring of the long-term outcome is important.^24^ Based on the results of a previous investigation, due to possible device degradation, patients with EGs should be offered life-long surveillance,^18^ and the Nellix device is no exception. Although Nellix has been withdrawn from the market, there are numerous patients with this type of EG. Due to its unpredictable performance in the medium and long term, these patients should be recommended enhanced life-long surveillance every 6 months.^21,24^ Any suspicious conditions during the follow-up must be taken seriously and explantation should be considered.

This article is among the first to report on the possible mechanisms for EVAS failure and, in particular, on the endobag polymer instability. The findings were similar for all the devices studied. Although in vivo devices are exposed to different extents of physiological stress it would be possible to expect the same alterations at least on part of other Nellix devices, given the manufacturing process and the polymer compounds are the same. It is of note that the results of this study are limited by the constraints of its experimental design. First, we have described the defects, but we could not demonstrate direct causality between these findings and the instability of the device. The revealed polymer changes, however, suggest our concept logical. Second, we analyzed the explanted material, but we were unable to assess the imaging and extensive clinical data to make definite conclusions on other possible reasons for failure. Ideally, such an analysis would require a separate study with modeling based on the anatomic particularities. Finally, we tried to simulate the intra-aneurysmal conditions during the dry phase, which can, apparently, vary in vivo. Although the present findings are reliable further analysis in a model setting would be beneficial.

## Conclusion

Despite its hydrophilic properties, the polymer can lose weight and volume upon dehydration. The polymer deteriorates and cannot retain the shape of the aneurysm. Structural degradation of the polymer within the endobags could lead to the instability of the device, with subsequent development of endoleaks or migration.
